# The Localisation of Kidney Tuberculosis

**Published:** 1899-01-28

**Authors:** 


### THE LOCALISATION OF KIDNEY TUBERCULOSIS.

IN the November number of the *Bulletin of the Johns Hopkins Hospital* there is an account by Dr. Edward Reynolds of a method of diagnosis of the condition of each kidney in suspected tuberculosis by means of the inoculation of the separated sediments into guinea pigs. It is only of late years that we have known that tuberculosis can be primary in the kidney, and the great mass of the profession has not yet realised that the disease is often localised for many years in one kidney before invading the rest of the urinary tract, nor is it fully recognised that when so localised the patient may be restored to perfect health if such a kidney is removed by nephrectomy. When, however, these things are understood, we see the immense importance of being able to diagnose not merely the presence of tuberculosis of the kidney, but of being able to say which kidney is affected. So far as the ordinary symptomatology goes the symptoms of tuberculous kidney are those of inflammation of that organ, and the cause of the inflammation has to be ascertained by other means, of which the recognition of the bacillus naturally suggests itself as the most important. But while the positive recognition of the bacillus is, of course, definite, its apparent absence says nothing. The inoculation test is, however, one of considerable certainty; and as it is possible in the case of women to catheterise each ureter separately, it becomes possible in such cases by inoculation of the sediment into guinea pigs to make sure which kidney is diseased. A good case is given in which this mode of diagnosis was resorted to, and in which, after operation, the patient made a satisfactory recovery.

